# Supplementary material for: Nutritional status and associated factors among adult patients with tuberculosis in public hospitals of Sidama Region, Ethiopia
Source: PLOS Glob Public Health. 2026 Jan 7;6(1):e0005714. doi: 10.1371/journal.pgph.0005714 (PMC12779151; doi:10.1371/journal.pgph.0005714)
Supplement: Attachment — Submitted filename: Response to Reviewers.docx [file pgph.0005714.s002.docx]

**Date: 20 November 2025**

# **Authors Response to Reviewers**

#### Manuscript Id: PGPH-D-25-02918

**Manuscript Title:** Nutritional Status and Associated Factors among Adult Patients with Tuberculosis in Public Hospitals of Sidama Region, Ethiopia

| S.No | Reviewer 1 Comments | Authors Response | Line No &Page No |
| --- | --- | --- | --- |
| #1 | **MAJOR COMMENTS**  The modelling approach is inconsistent with the aim of the study, which was to identify determinants of undernutrition and anemia. Unless the overall aim was to derive a risk prediction algorithm, it is not appropriate to simply put all variables with an arbitrary p<0.25 in univariable analysis into a single multivariable model and see which ones come out as being associated. Please see here for further discussion on this topic: <https://doi.org/10.1093/ije/dyaa213>. I would suggest the authors redo the analysis using a DAG to inform the choice of confounders. We should be trying to move away from Table 2 approaches in our research: <https://pubmed.ncbi.nlm.nih.gov/23371353/> | **Dear Reviewer,**  Thank you for your insightful comment regarding the modeling approach and your suggestion to use a Directed Acyclic Graph (DAG) to inform the choice of confounders. We appreciate the references provided and the emphasis on moving away from traditional "Table 2" approaches in epidemiological research.  While we acknowledge the increasing adoption of DAGs as a robust tool for causal inference and confounder selection, our study was specifically designed to explore potential determinants of undernutrition and anemia using an exploratory approach. The primary aim was not to establish causality but rather to identify significant associations that could inform future research or interventions in this area. Given this aim, we prioritized a pragmatic approach using p-values from bivariable analysis as a screening tool to identify candidate variables for the multivariable model. This method, while not without its limitations, is widely accepted in exploratory epidemiological studies and aligns with the objectives of our research.  We selected the p < 0.25 threshold for bivariable analysis as it is a commonly used criterion to ensure that potentially relevant variables were not excluded prematurely. This approach has been used in many similar studies, as it balances the need for parsimony with the risk of omitting important predictors. Furthermore, our multivariable model was carefully constructed to account for potential collinearity and overfitting, ensuring that the results are robust and interpretable within the scope of our study.  While we agree that DAGs can provide a more structured and theoretically informed approach to confounder selection, the construction of a DAG requires comprehensive prior knowledge of causal relationships between all variables, which was not feasible in this context given the exploratory nature of our study and the available data. Nevertheless, we consider this suggestion valuable and will explore the use of DAGs in future research to strengthen our methodological framework.  In conclusion, we believe that our current approach is consistent with the exploratory aim of the study and provides valuable insights into the determinants of undernutrition and anemia. We respectfully request that our modeling approach remain unchanged, as it is appropriate for the objectives and scope of this study.  In addition, we utilized Modified Poisson Regression to assess associations because it directly estimates prevalence ratios, providing clearer insights into the effect of predictors on the probability of events occurring. This approach is particularly beneficial in public health nutrition, where understanding prevalence ratios is often more relevant than odds ratios.  Thank you again for your thoughtful feedback, which has prompted us to reflect on the methodological choices and potential improvements for future work. |  |
| Minor Comments | | | |
| #3 | **Introduction**  Should be updated to reflect new WHO guidelines (<https://www.who.int/publications/i/item/9789240111967>) and more recent evidence on BMI and TB (<https://doi.org/10.1093/ije/dyaf154>) | Dear Review, after carefully reviewing the recommended references, considering they are updated references, we incorporated them into revised manuscript. | Line 45, 380, 437, 591; Page 3, 25; Reference 1, Reference 56 |
|  | **Introduction**  Last paragraph does not effectively justify study rationale. The phrase "This gap underscores the need for further investigation to thoroughly understand the comprehensive effectiveness of the TB treatment program" is not relevant to the current manuscript. The aims need to be made more explicitly clear - estimate the prevalence of undernutrition and anemia, and identify variables associated with these. | **Dear Reviewer,**   - The specified last two sentences were rephrased to clearly justify the study rationale. | Line 90-92; Page 5 |
|  | **Methods**  More detail is required on the inclusion/exclusion criteria, sampling methods, and participants. Presumably these were people with TB being treated as outpatients? Were they all recently diagnosed, or was it a cross section of anyone on TB treatment? I see in the results this is presented (87% newly diagnosed), but needs to be much clearer and more justified in the methods (e.g. recruiting people already on treatment will affect results, for various reasons). | Dear Reviewer,   - The detailed criteria used to include and exclude study participants were incorporated into the ‘Study Population and Eligibility Criteria’ subsection of the methodology. - As shown in the methodology and results sections of our manuscript, we included patients who met the inclusion criteria from among TB patients attending the TB clinics of the selected hospitals for outpatient treatment. We reported that 86.6% of the TB patients were newly diagnosed simply to indicate their treatment status; the remaining patients were on re-treatment. | Line 116-121; Page 6 |
|  | **Methods**  Please provide more detail on the way HFIAS is used to defined food secure or not, or add a reference. | Dear Reviewer, a reference is added as recommended. | Line 163-166; Page 8 |
|  | **Methods**  What justification is there for the arbitrary higher knowledge score of 13/16 rather than using this as a continuous variable? - Only DS-TB, or also DR-TB? - The statistical methods section needs more detail. | Dear Reviewer   - We used knowledge as a categorical variable considering the nature of the model used to assess association, to mitigate the influence of outliers, and to facilitate easier interpretation and understanding of our findings by non-technical audiences. - In addition, since our study tool has no pre-defined cut-off points, using ≥80% of the total score to define high knowledge is a widely acceptable and interpretable standard. - During data collection, we excluded drug-resistant TB patients from our study. - Data management and analysis subsection of the manuscript was more detailed as commented. |  |
|  | **Results:** Should be made more readable. There is no need to write out in full text numbers like "three hundred thirty-three". The narrative should be brief, with references made to the tables. | Daer Reviewer, the given comment was applied throughout the result section of the manuscript. | Line 221-232; Page 10 |
|  | **Results**: For the wealthy index I would suggest lower, medium, or higher rather than poor, medium, or rich | Dear reviewer, we consider the presentation of the wealth index categories as either ‘poor, medium, and rich’ or ‘lower, medium, and higher’ to be equivalent, and we have revised the manuscript as per your comment. | Line 232; Page 10 and Table 1, Page 12 |
|  | **Results**: Severe, moderate, and mild undernutrition are not defined. | Dear Reviewer, the definitions of mild, moderate, and severe undernutrition were provided in the ‘Study Variables’ subsection of the methodology. The prevalence of each category was reported in the ‘Undernutrition among TB patients’ subsection of the results and illustrated in the corresponding figure. | Line 138-142, Page 7, and L 277-279, Page 15 |
| Reviewer 2 Comments | | | |
| S.No | **Reviewer 2 Comments** | **Authors Response** | **Line No &Page No** |
| #1 | **Person-centered language**  I’d recommend replacing the term TB patient with person with tuberculosis (PWTB) throughout. This aligns with WHO guidance and helps convey a more person-centered and respectful tone and one expected by TB civil society. | Dear Reviewer, the raised issues have been corrected throughout the document as you commented. | Line 18; Page 2-22 |
| #2 | **Clarifying the definition of undernutrition**  The study defines undernutrition as BMI <18.5 kg/m², but it would be helpful to state explicitly that this operational definition corresponds to underweight. Providing additional granularity—such as severe (<16), moderate (16–16.9), and mild (17–18.4)—would parallel your nuanced classification of anemia and make the findings more interpretable. These cutoffs correspond to those recommended by the WHO | Dear Reviewer, the operational definitions of mild, moderate, and severe undernutrition were incorporated into ‘Study Variables’ subsection of the methodology as commented | Line 138-142, Page 7 |
| #3 | **Recognizing hidden forms of malnutrition**  It might strengthen the discussion to note that BMI alone may underestimate the true burden of nutritional deficiency. Many individuals with “normal” BMI likely have unmeasured micronutrient deficiencies, particularly given the low dietary diversity you document. A brief mention of this limitation would add nuance and align your discussion with the broader literature on “hidden hunger.” | Dear Reviewer,  Thank you for raising this important issue. After discussing it with all the authors, the issue you raised is addressed in the discussion section of the manuscript. | L 369-378, Page 21 |
